# Incidence of collagen-induced arthritis is elevated by a high-fat diet without influencing body weight in mice

**DOI:** 10.1136/rmdopen-2023-003869

**Published:** 2024-04-04

**Authors:** Jianhui Liang, Kuangyang Yang, Yanni Shen, Xiao Peng, Hao Tan, Lichu Liu, Qian Xie, Yan Wang

**Affiliations:** 1 Shenzhen Institutes of Advanced Technology Chinese Academy of Sciences, Shenzhen, Guangdong, China; 2 Foshan Hospital of Traditional Chinese Medicine, Foshan, China; 3 Department of Orthopaedics, Shenzhen University General Hospital, Shenzhen, China

**Keywords:** Arthritis, Rheumatoid, Rheumatoid Factor, Outcome Assessment, Health Care

Obesity is recognised as a risk factor for triggering rheumatoid arthritis (RA), and it can worsen joint deformities^1^ and diminish the quality of life in patients with RA.^2^ The reduction of body weight in obese individuals is believed to alleviate RA symptoms.^3^ Body mass index (BMI) serves as the primary standard for evaluating obesity.^4^ An increase in BMI by 1 SD notably elevates the incidence rate of RA, suggesting a causal link between higher BMI and an increased risk of developing RA.^5^

The association between BMI and obesity is straightforward, as a higher BMI typically indicates a greater risk of obesity.^4^ Obesity is clinically defined as having a BMI of 30 kg/m^2^ or greater.^4^ Here, we established collagen-induced arthritis (CIA) models in mice using both regular and high-fat diets (HFDs) to see if HFD can induce severe RA symptoms in mice. CIA mice develop arthritis symptoms, including joint swelling, redness and stiffness, mirroring the clinical features of human RA.^6^ Therefore, the CIA model has become the prevailing animal model for RA research.

Our observations indicated that mice in the HFD sham group exhibited significantly higher body weights compared with regular diet group, in line with previous reports.^7^ However, the HFD CIA model group did not show a significant difference in body weight compared with mice on a regular diet (figure 1A). Interestingly, although the HFD did not induce higher body weight in the CIA model, arthritis incidence was notably higher in HFD-fed mice starting from day 30 when compared with those on regular diets (figure 1B). Furthermore, the HFD sham group displayed significantly higher levels of cholesterol (CHO) and high-density lipoprotein (HDL) compared with the regular diet sham group, whereas the HFD CIA group exhibited similar serum levels of CHO, HDL, low-density lipoprotein and triglycerides compared with the regular diet CIA group (figure 1C).

**Figure 1:**
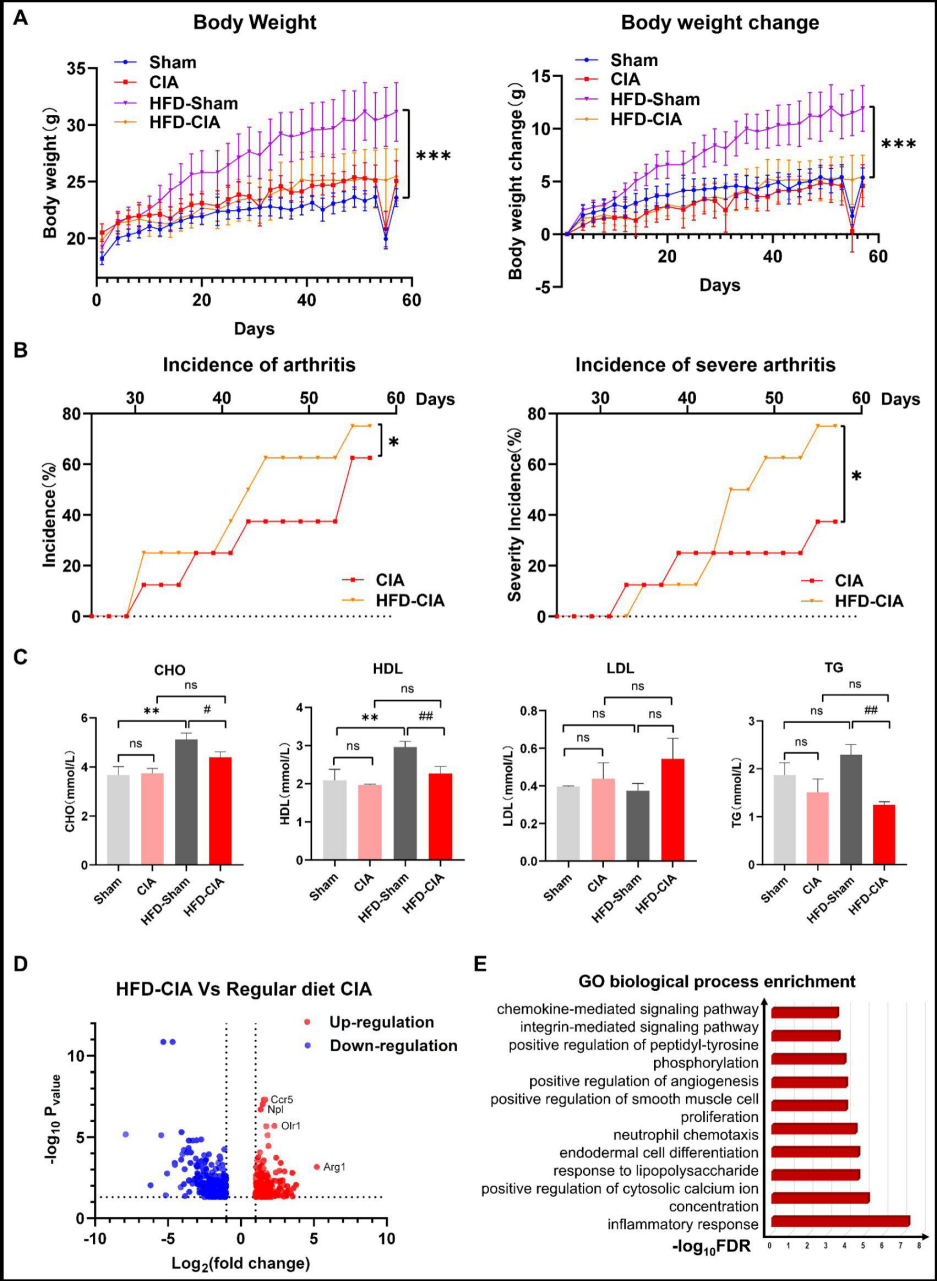
High-fat diet (HFD) did not lead to an increase in body weight but significantly exacerbated the arthritis incidence in the CIA model. (A) Body weights of mice were tracked over a 60-day period. The data represent the mean±SEM, with n=6 per group, and were analysed using analysis of variance (ANOVA) followed by Bonferroni’s post-tests. ***p<0.001 compared with the sham group. (B) The incidence of arthritis in mice was observed over a 60-day period. The data represent the mean±SEM, with n=6 per group, and were analysed using ANOVA followed by Bonferroni’s post-tests. *p<0.05 compared with CIA mice on regular diets. (C) Cholesterol (CHO), high-density lipoprotein (HDL), low-density lipoprotein (LDL) and triglyceride (TG) levels were measured at the end of the experiment. The data represent the mean±SEM, with n=6 per group, and were analysed using ANOVA followed by Bonferroni’s post-tests. **p<0.01 compared with the sham group on regular diets, whereas ^#^p<0.05 and ^##^p<0.01 compared with the sham group on HFD. (D) A volcano plot illustrates differentially expressed genes between CIA mice on regular diets and HFD (n=3 biologically independent cell samples). Significant genes were identified using Cuffdiff. Red dots represent upregulated transcripts, while blue dots represent downregulated transcripts. (E) GO biological process enrichment analysis of key targets. CIA, collagen-induced arthritis; GO, Gene Ontology.

To gain insights into the mechanisms behind the exacerbation of arthritis by HFD, we collected synovial tissue and performed RNA sequencing. Our RNA-Seq analysis revealed that 217 genes in the HFD CIA model were significantly upregulated, while 354 genes were significantly downregulated compared with the regular diet CIA model (figure 1D). Gene Ontology analysis demonstrated the activation of pathways related to inflammatory responses in the HFD CIA model (figure 1E), indicating that HFD leads to inflammation in synovial tissue. Our tissue immunohistochemistry experiments revealed higher expression of the inflammatory representative factors IL-1b and TNF-α in HFD-CIA compared with CIA in the regular diet (online supplemental figure S1). Interestingly, CIA model mice on HFD did not exhibit more severe subchondral erosion (online supplemental figure S2) or cartilage damage (online supplemental figure S3) when compared with CIA model mice on a regular diet.

10.1136/rmdopen-2023-003869.supp1 Supplementary data

In summary, HFD did not lead to an increase in body weight or greater subchondral erosion and cartilage damage in the CIA model. However, it significantly exacerbated the rheumatic index. Given that HFD has been reported to induce chronic inflammation,^8^ which aligns with the activation of inflammatory pathways found in our sequencing analysis. We conclude here that a single BMI measurement may not be sufficient as an indicator of RA disease risk. It is advisable to consider the adoption of a HFD and its fat content when assessing the risk of RA.

## Data Availability

The RNA-Seq data were deposited in GEO (GSE260460).
