# Supplementary material for: Incidence of collagen-induced arthritis is elevated by a high-fat diet without influencing body weight in mice
Source: RMD Open. 2024 Apr 4;10(2):e003869. doi: 10.1136/rmdopen-2023-003869 (PMC11002344; doi:10.1136/rmdopen-2023-003869)
Supplement: Supplementary data [file rmdopen-2023-003869supp001.pdf]

Supplementary materials

The incidence of collagen-induced arthritis is elevated by a high-fat diet without influencing body weight in mice

Jianhui Liang[a,1], Kuangyang Yang[b,1], Yanni Shen[a,1], Xiao Peng[b], Hao Tan[b], Lichu Liu[b], Qian Xie[a,c,*], Yan Wang[a,*]

[a] Center for Translational Medicine Research and Development, Shenzhen Institute of Advanced Technology, Chinese Academy of Sciences, Shenzhen, 518055, China

[b] Foshan Hospital of Traditional Chinese Medicine, Foshan 528000, China

[c] Department of orthopaedics, Shenzhen University General Hospital, Shenzhen, 518055, China

[1] These authors contributed equally to this work.

[*]**Correspondences:** Dr Qian Xie, Center for Translation Medicine Research and Development, Shenzhen Institutes of Advanced Technology, Chinese Academy of Sciences, Shenzhen 518055, China; Department of orthopaedics, Shenzhen University General Hospital, Shenzhen, 518055, China, Tel.: +86-755-2641-7985; E-mail: qian.xie@szu.edu.cn; Dr. Yan Wang, Center for Translation Medicine Research and Development, Shenzhen Institutes of Advanced Technology, Chinese Academy of Sciences, Shenzhen 518055, China; Tel.: +86-755-2641-7985; E-mail:yan.wang@siat.ac.cn .

Liang J, *et al. RMD Open* 2024; 10:e003869. doi: 10.1136/rmdopen-2023-003869

Supplementary materials

Supplement Figure 1. High-fat diet CIA mice exhibited more inflammation in synovium compared to CIA RA mice on regular diets

Supplement Figure 2. High-fat diet CIA mice did not display more severe subchonral damage compared to CIA RA mice on regular diets

Supplement Figure 3. High-fat diet CIA mice did not display more severe cartilage damage compared to CIA RA mice on regular diets

Materials and methods

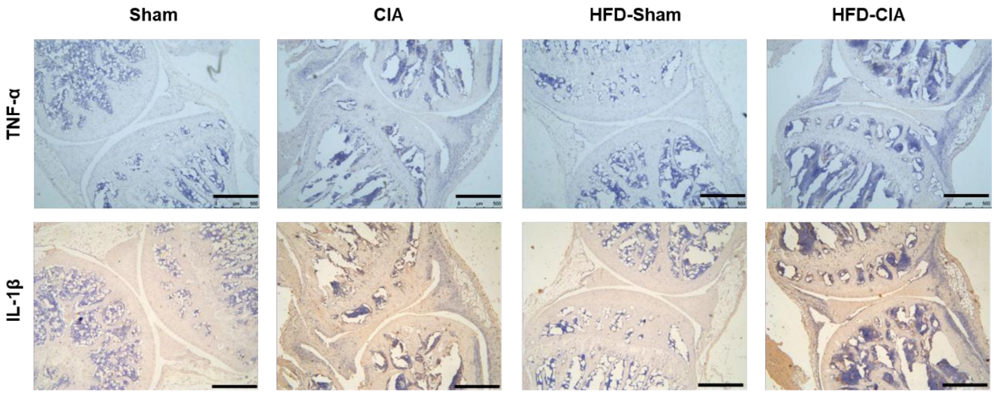

Supplement Figure 1. High-fat diet CIA mice exhibited more inflammation in synovium compared to CIA RA mice on regular diets. Immunohistochemical staining of TNF-α (upper) and IL-1b (down) in joint (scale bar: 500 μm).

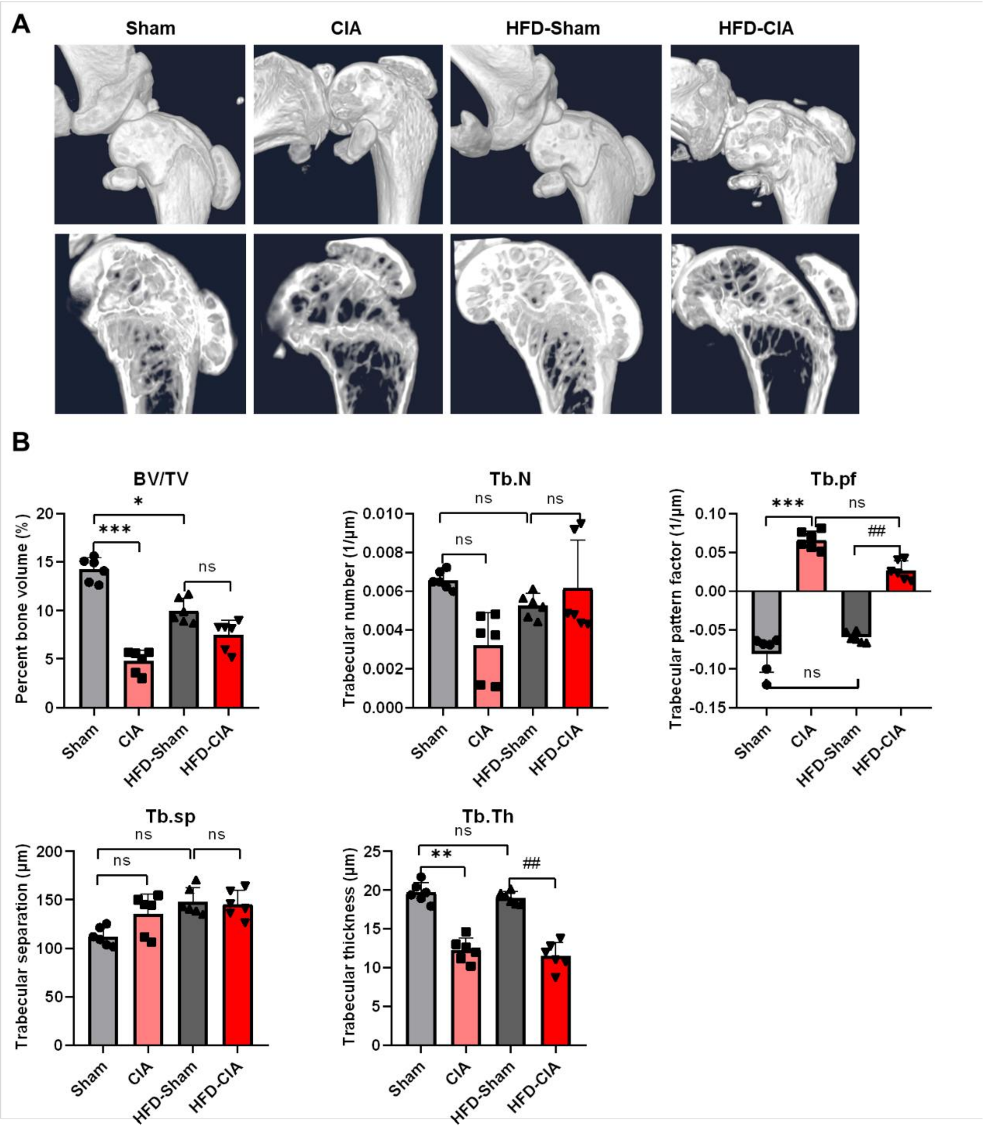

Figure S2. High-fat diet CIA mice did not display more severe subchonral damage compared to CIA RA mice on regular diets. (A) Mice underwent micro-CT 2D imaging and 3D reconstructions of the femur 60 days after collagen injection. **(B)** Various bone parameters, including bone volume over tissue volume (BV/TV), bone trabecular thickness (Tb.Th), trabecular separation (Tb.Sp), number of trabeculae (Tb.N), and trabecular pattern factor (Tb.Pf), were assessed 60 days after collagen injection. The data represent the mean ± SEM, with n = 6 per group, and were analyzed using ANOVA followed by Bonferroni's post-tests. $^{*}p < 0.05$, $^{**}p < 0.01$, $^{***}p < 0.001$ compared to the sham group on regular diets, whereas $^{\#\#}p < 0.01$ compared to the sham group on HFD.

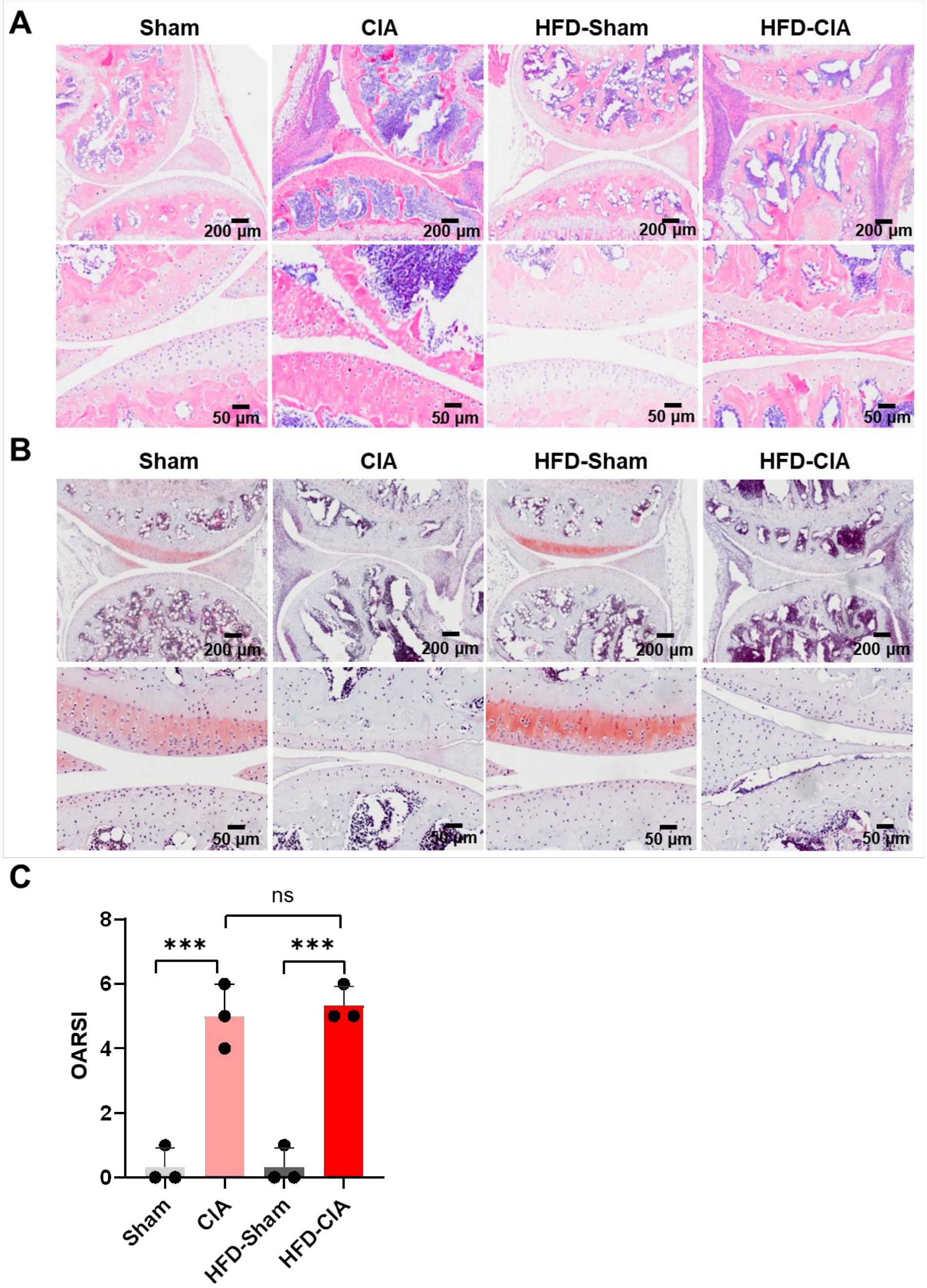

Figure S3. High-fat diet CIA mice did not display more severe cartilage damage compared to CIA RA mice on regular diets. Joint tissues were obtained from the Sham, CIA, HFD-Sham, and HFD-CIA groups on day 60 and were stained with **(A)** hematoxylin and eosin (H&E) and **(B)** safranin O to assess the extent of inflammation and cartilage damage. **(C)** The joints were scored histologically using the Osteoarthritis Research Society International (OARSI) scoring system.

Materials and methods

CIA model

Thirty-two male DBA/1 mice, aged between 7 and 8 weeks, were purchased from Vital River in Beijing, China, and housed in the SIAT animal housing center under controlled temperature conditions. The mice were randomly divided into four groups by using a computer based random order generator (https://www.randomizer.org/), a sham group and a CIA group on regular diets and a sham group and a CIA group on HFD, with eight mice in each group. The HFD group commenced one week prior to the initial immunization and was consistently maintained on a HFD comprising 45% of high-fat until the conclusion of the experiment. The 45% high-fat feed, formulated with the reference composition D12451, includes the following ingredients: Casein Lactic, 30 MESH: 200.00 g, Cystine, L: 3.00 g, Sucrose: 176.80 g, LODEX 10: 100.00 g, Lard: 177.50 g, Soybean Oil: 25.00 g, STARCH, Corn: 72.80 g, SOLKA FLOC: 50.00 g, S10026B: 50.00 g, Choline Bitartrate: 2.00 g, and V10001C: 1.00 g.For the initial immunization, an injection emulsion composed of a mixture of equal volumes of bovine type II collagen (2mg/mL in 0.05M acetic acid, Chondrex, USA) and Complete Freund's Adjuvant (4mg/mL, Chondrex, USA) was subcutaneously injected into the root of each rat's tail at a volume of 200 μL. After one week, 100 μL of emulsion mixed with equal volumes of bovine type II collagen and Incomplete Freund's Adjuvant (4mg/mL, Chondrex, USA) were again subcutaneously injected into the tail. Mice in the sham group were injected with an equal volume of saline. The injection order was randomized. Initially, we initiated the experiment with 8 mice in each group. However, during the experiment, two mice in both the CIA group and HFD-CIA group unfortunately passed away. Consequently, our final dataset consists of 6 mice in each group. After 60 days, all CIA mice experienced severe systemic multi-joint inflammation. The mice were randamly euthanized using an overdose of anesthesia. In the final analysis, each of the six mice in every group underwent weight measurement and their seurm was collected for ELISA to moniter the concentration of CHO, TG, HDL and LDL. Moreover, MicroCT scanning was performed on six left knees of each

mouse. For further analysis, the three right knees of each mouse were utilized for RNA sequencing, while the remaining three right knees underwent slicing. The Institutional Animal Care and Use Committee (IACUC) at SIAT approved the study, with registration number SIAT-IACUC-210226-YGS-WY-A1644.

Mirco-CT

To conduct micro-CT imaging of the rat knee joints, the joints were initially fixed with 4% neutral formalin for two days. Next, they were placed in a 34mm scanning tube and scanned using a Scanco Microct, μCT100 machine from Switzerland, with a resolution of 18 μm and a voltage/current value of 55kV/200μA. Subsequently, CTan and CTvol software (Bruker, Belgium) were used to analyze and obtain the resulting images and reconstruction data.

Safranin O/ Fast Green Staining

Following the micro-CT imaging, the joints underwent decalcification with 10% EDTA at pH 7.4 for 30 days, after which they were embedded in paraffin and sliced into 6-μm-thick sections. The sections were then dewaxed with xylene, gradually rehydrated with ethanol, and stained with Safranin O/ Fast Green (sigma, USA) for visualization of cartilage.

RNA-seq library construction and sequencing

To perform RNA-seq profiling, the joints were dissected on an ice bath to collect the synovial tissue, which was then rinsed with pre-cooled PBSE (a PBS buffer containing 2 mM EGTA) prior to experimentation. Subsequently, the cells were collected and sent to the BGI Genomics institution for RNA extraction and bulk mRNA sequencing. Libraries were constructed according to the manufacturer’s instructions (Illumina, San Diego, CA, USA), and paired-end sequencing was conducted using the Illumina Hiseq2000 sequencer (Illumina, USA). Both library construction and RNA-seq were conducted at BGI. The raw sequencing reads (fastq) quality was checked using SOAPnuke (v1.5.2), and alignment was performed by Bowtie2 (v2.2.5). The expression

level of genes was calculated using RSEM (v1.2.12), while DESeq2 (v1.4.5) was used for differential expression analysis with a *p*-value < 0.05.

Statistical Analysis

Graph Pad Software 9.0 (San Diego, CA, USA) was used to perform one-way ANOVA test to determine the statistical significance. The results were presented as the means ± SEM, and the *p*-value less than 0.05 were considered significant.
